# The genome sequence of the Bordered Straw, *Heliothis peltigera *(Denis & Schiffermüller) 1775

**DOI:** 10.12688/wellcomeopenres.21221.1

**Published:** 2024-04-15

**Authors:** Denise C. Wawman, Liam M. Crowley, University of Oxford and Wytham Woods Genome Acquisition Lab, Darwin Tree of Life Barcoding collective, Wellcome Sanger Institute Tree of Life Management, Samples and Laboratory team, Wellcome Sanger Institute Scientific Operations: Sequencing Operations, Wellcome Sanger Institute Tree of Life Core Informatics team, Tree of Life Core Informatics collective, Darwin Tree of Life Consortium

**Affiliations:** 1 Department of Biology, University of Oxford, Oxford, England, UK

**Keywords:** Heliothis peltigera, Bordered Straw moth, genome sequence, chromosomal, Lepidoptera

## Abstract

We present a genome assembly from an individual male *Heliothis peltigera* (the Bordered Straw; Arthropoda; Insecta; Lepidoptera; Noctuidae). The genome sequence is 332.8 megabases in span. Most of the assembly is scaffolded into 31 chromosomal pseudomolecules, including the Z sex chromosome. The mitochondrial genome has also been assembled and is 15.3 kilobases in length. Gene annotation of this assembly on Ensembl identified 17,114 protein coding genes.

## Species taxonomy

Eukaryota; Opisthokonta; Metazoa; Eumetazoa; Bilateria; Protostomia; Ecdysozoa; Panarthropoda; Arthropoda; Mandibulata; Pancrustacea; Hexapoda; Insecta; Dicondylia; Pterygota; Neoptera; Endopterygota; Amphiesmenoptera; Lepidoptera; Glossata; Neolepidoptera; Heteroneura; Ditrysia; Obtectomera; Noctuoidea; Noctuidae; Heliothinae; *Heliothis*; *Heliothis peltigera* (Denis & Schiffermüller) 1775 (NCBI:txid542982).

## Background

The Bordered Straw *Heliothis peltigera* is a Noctuid moth, but although it has the characteristic thick body and wing-shape seen in this family, it is brighter coloured than most of its dull brown cousins, with a ground colour ranging from straw-yellow, early in the season, to darker orange-brown, as increasing temperatures in the larval stages causes the adults to become darker ( Waring *et al.*, 2017). It is usually more distinctly marked than similar species with a bold grey kidney mark and diagnostic dark diffuse blotches on the leading edge of the forewing near the kidney and adjacent to the darker band on the outer edge: the similar Scarce Bordered Straw or Old World Bollworm *Helicoverpa armigera* lacks both of these diagnostic markings, and Eastern Bordered Straw *Heliothis nubigera* has only a smaller more diffuse marking near the kidney, with a paler ground colour and three or more distinct small dots near the outer edge ( Skinner & Wilson, 2009; Waring *et al.*, 2017).

Within the United Kingdom *Heliothis peltigera* is considered to be an immigrant species ( Waring *et al.*, 2017), but it is common in tropical and subtropical areas of Africa, where it is a major agricultural pest and, with climate change, is spreading north into Europe ( Ragionieri *et al.*, 2017). The larvae cause damage to crops such as safflower *Carthamus tinctorius*, tobacco *Nicotiana tabacum*, grapevine *Vitus* spp., cotton *Gossypium* spp., stone and citrus fruit trees, fodder crops, medicinal and ornamental plants ( Dunkelblum & Kehat, 1992). Consequently, the synthesis and production of pheromones by *H. peltigera* ( Altstein, 2004; Ragionieri *et al.*, 2017) and the moths’ response to them ( Dunkelblum & Kehat, 1992) have been the subject of intense research. More recently molecular methods have been used to identify a strain of *Helicoverpa armigera* single nucleopolyhedrovirus (HearNPV-TR), which infects *H. peltigera*, and is similar to other viruses which have been used for the biological control of other insect pests ( Eroglu *et al.*, 2020).

We present a chromosomally complete genome sequence for *Heliothis peltigera*, based on one specimen collected using a mercury vapour light trap in a rural garden in the hamlet of Bratton, near Minehead, in Somerset, as part of the Darwin Tree of Life Project.

## Genome sequence report

**Figure 1.**
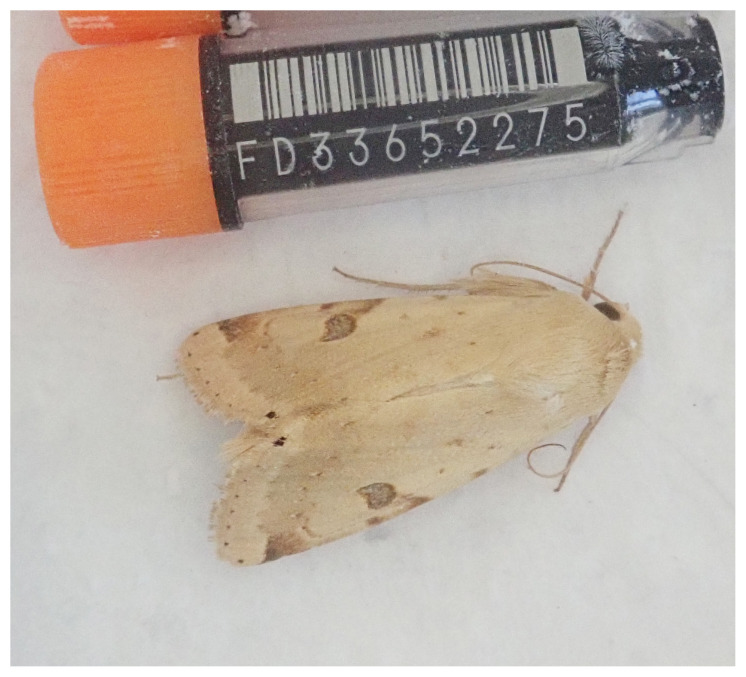
Photograph of the *Heliothis peltigera* (ilHelPelt1) specimen used for genome sequencing.

The genome was sequenced from a male *Heliothis peltigera* ( Figure 1) collected from Bratton, Somerset, UK (51.20, –3.51). A total of 75-fold coverage in Pacific Biosciences single-molecule HiFi long reads was generated. Primary assembly contigs were scaffolded with chromosome conformation Hi-C data. Manual assembly curation corrected 4 missing joins or mis-joins, increasing the scaffold number by 3.23%.

**Figure 2.**
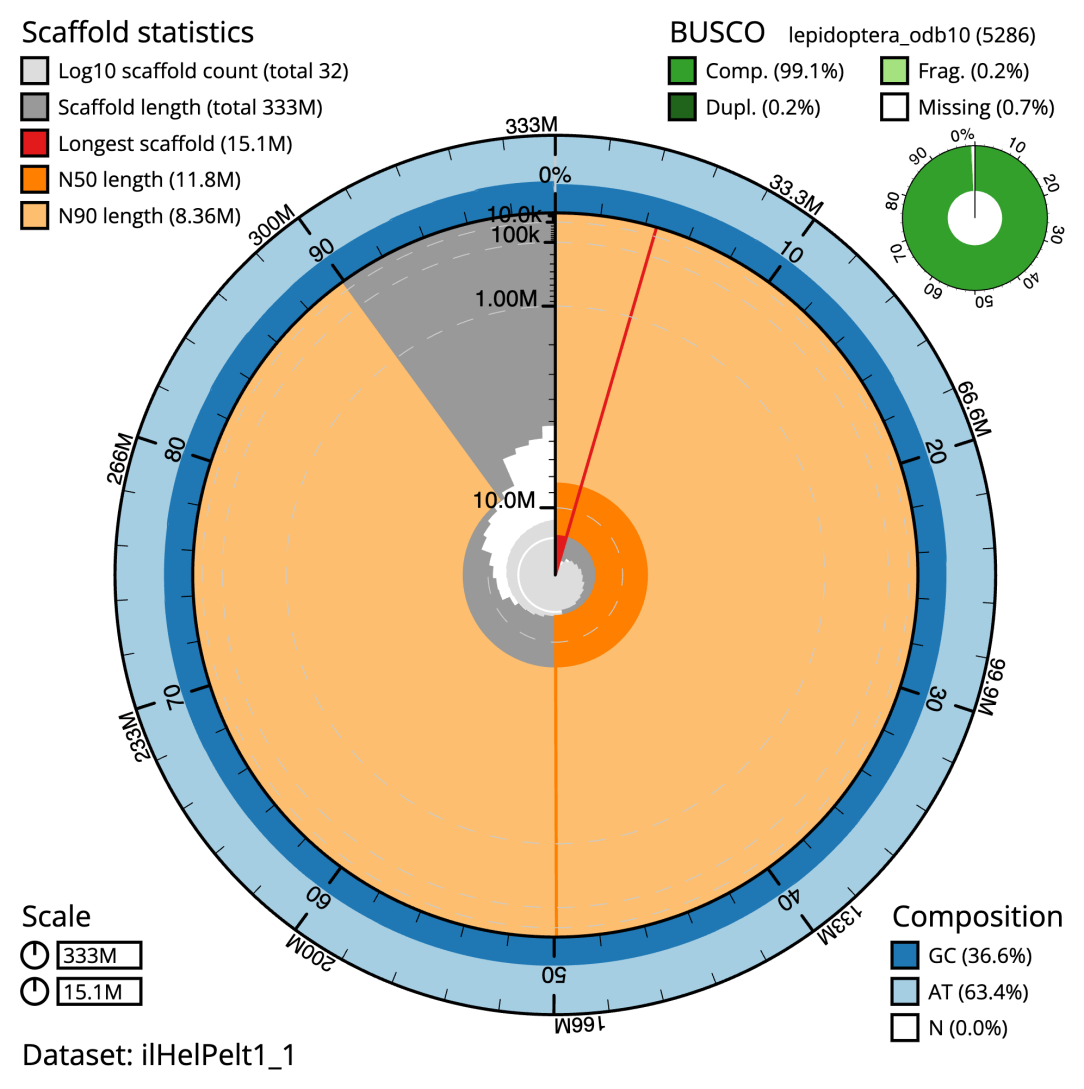
Genome assembly of *Heliothis peltigera*, ilHelPelt1.1: metrics. The BlobToolKit snail plot shows N50 metrics and BUSCO gene completeness. The main plot is divided into 1,000 size-ordered bins around the circumference with each bin representing 0.1% of the 332,843,978 bp assembly. The distribution of scaffold lengths is shown in dark grey with the plot radius scaled to the longest scaffold present in the assembly (15,076,996 bp, shown in red). Orange and pale-orange arcs show the N50 and N90 scaffold lengths (11,813,164 and 8,357,070 bp), respectively. The pale grey spiral shows the cumulative scaffold count on a log scale with white scale lines showing successive orders of magnitude. The blue and pale-blue area around the outside of the plot shows the distribution of GC, AT and N percentages in the same bins as the inner plot. A summary of complete, fragmented, duplicated and missing BUSCO genes in the lepidoptera_odb10 set is shown in the top right. An interactive version of this figure is available at https://blobtoolkit.genomehubs.org/view/ilHelPelt1_1/dataset/ilHelPelt1_1/snail.

**Figure 3.**
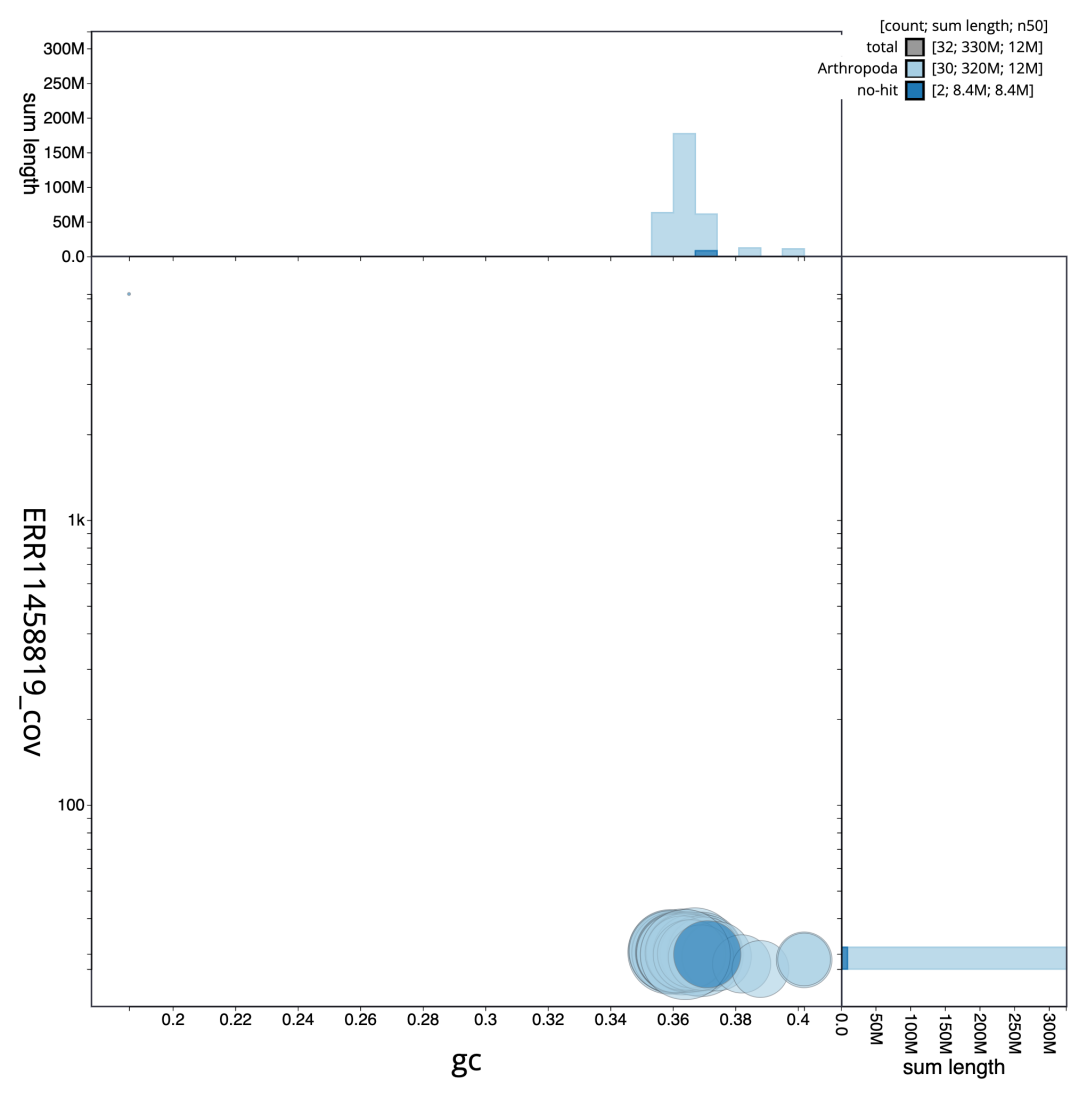
Genome assembly of *Heliothis peltigera*, ilHelPelt1.1: BlobToolKit GC-coverage plot. Sequences are coloured by phylum. Circles are sized in proportion to sequence length. Histograms show the distribution of sequence length sum along each axis. An interactive version of this figure is available at https://blobtoolkit.genomehubs.org/view/ilHelPelt1_1/dataset/ilHelPelt1_1/blob.

**Figure 4.**
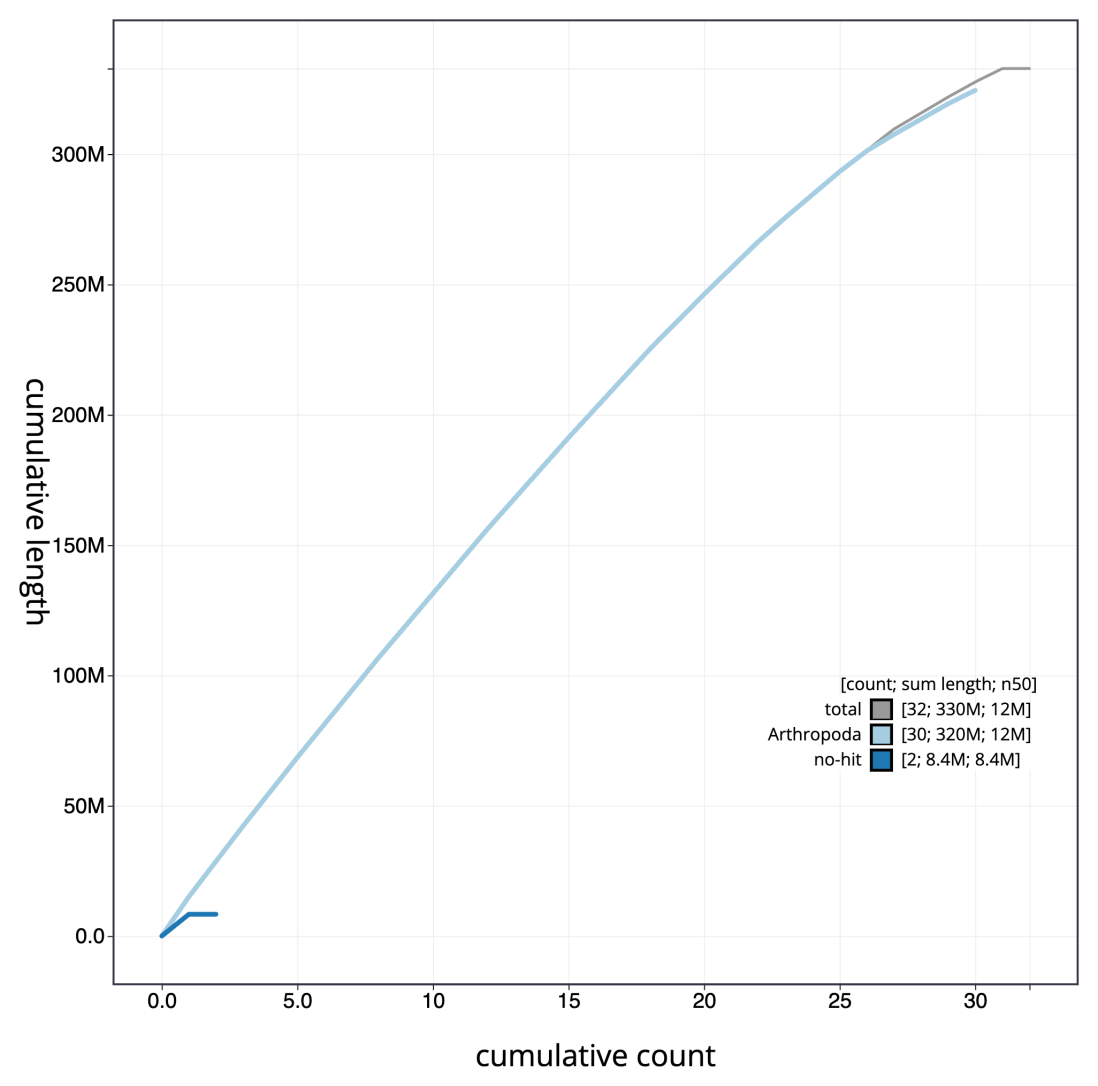
Genome assembly of *Heliothis peltigera*, ilHelPelt1.1: BlobToolKit cumulative sequence plot. The grey line shows cumulative length for all sequences. Coloured lines show cumulative lengths of sequences assigned to each phylum using the buscogenes taxrule. An interactive version of this figure is available at https://blobtoolkit.genomehubs.org/view/ilHelPelt1_1/dataset/ilHelPelt1_1/cumulative.

**Figure 5.**
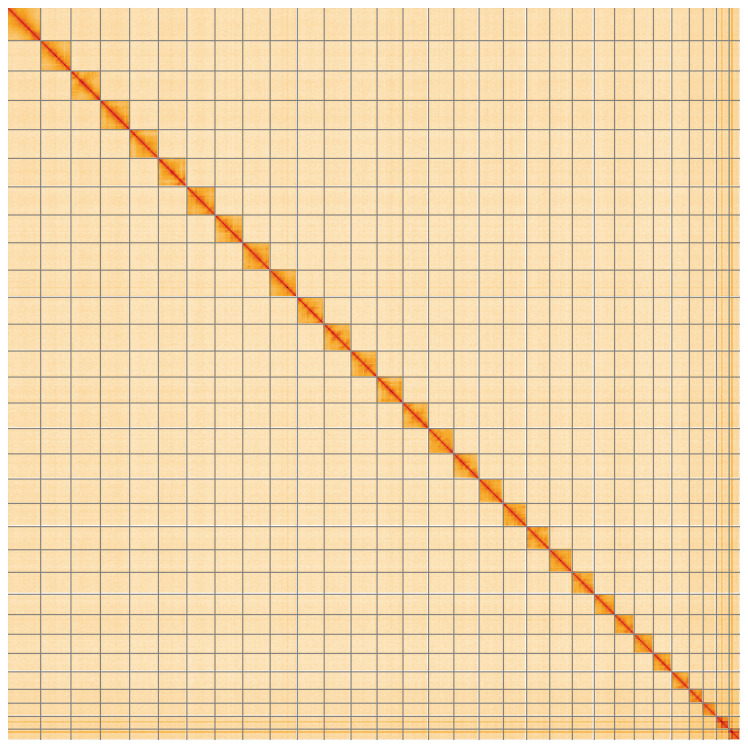
Genome assembly of *Heliothis peltigera*, ilHelPelt1.1: Hi-C contact map of the ilHelPelt1.1 assembly, visualised using HiGlass. Chromosomes are shown in order of size from left to right and top to bottom. An interactive version of this figure may be viewed at https://genome-note-higlass.tol.sanger.ac.uk/l/?d=YWF6i8BXRhmu_mJj5vyPDg.

The final assembly has a total length of 332.8 Mb in 31 sequence scaffolds with a scaffold N50 of 11.8 Mb ( Table 1). The snail plot in Figure 2 provides a summary of the assembly statistics, while the distribution of assembly scaffolds on GC proportion and coverage is shown in Figure 3. The cumulative assembly plot in Figure 4 shows curves for subsets of scaffolds assigned to different phyla. Most (99.99%) of the assembly sequence was assigned to 31 chromosomal-level scaffolds, representing 30 autosomes and the Z sex chromosome. Chromosome-scale scaffolds confirmed by the Hi-C data are named in order of size ( Figure 5; Table 2). The Z chromosome was identified based on synteny with *Craniophora ligustri* (GCA_905163465.1) ( Boyes *et al.*, 2023). While not fully phased, the assembly deposited is of one haplotype. Contigs corresponding to the second haplotype have also been deposited. The mitochondrial genome was also assembled and can be found as a contig within the multifasta file of the genome submission.

**Table 1.** Genome data for *Heliothis peltigera*, ilHelPelt1.1.

Project accession data		
Assembly identifier	ilHelPelt1.1	
Species	*Heliothis peltigera*	
Specimen	ilHelPelt1	
NCBI taxonomy ID	542982	
BioProject	PRJEB62179	
BioSample ID	SAMEA112226458	
Isolate information	ilHelPelt1, male: thorax (DNA sequencing), head (Hi-C scaffolding)	
Assembly metrics *		*Benchmark*
Consensus quality (QV)	68.1	*≥ 50*
*k*-mer completeness	100.0%	*≥ 95%*
BUSCO **	C:99.1%[S:98.9%,D:0.2%], F:0.2%,M:0.7%,n:5,286	*C ≥ 95%*
Percentage of assembly mapped to chromosomes	99.99%	*≥ 95%*
Sex chromosomes	ZZ	*localised homologous pairs*
Organelles	Mitochondrial genome: 15.3 kb	*complete single alleles*
Raw data accessions		
PacificBiosciences SEQUEL II	ERR11458819	
Hi-C Illumina	ERR11468748	
Genome assembly		
Assembly accession	GCA_958496145.1	
*Accession of alternate haplotype*	GCA_958496135.1	
Span (Mb)	332.8	
Number of contigs	56	
Contig N50 length (Mb)	9.2	
Number of scaffolds	31	
Scaffold N50 length (Mb)	11.8	
Longest scaffold (Mb)	15.08	
Genome annotation		
Number of protein-coding genes	17,114	
Number of gene transcripts	17,323	

* Assembly metric benchmarks are adapted from column VGP-2020 of “Table 1: Proposed standards and metrics for defining genome assembly quality” from Rhie *et al.* (2021). ** BUSCO scores based on the lepidoptera_odb10 BUSCO set using version 5.3.2. C = complete [S = single copy, D = duplicated], F = fragmented, M = missing, n = number of orthologues in comparison. A full set of BUSCO scores is available at https://blobtoolkit.genomehubs.org/view/ilHelPelt1_1/dataset/ilHelPelt1_1/busco.

**Table 2.** Chromosomal pseudomolecules in the genome assembly of *Heliothis peltigera*, ilHelPelt1.

INSDC accession	Chromosome	Length (Mb)	GC%
OY292322.1	1	13.75	36.5
OY292323.1	2	13.4	36.5
OY292324.1	3	13.25	37.0
OY292325.1	4	13.06	36.0
OY292326.1	5	12.89	36.5
OY292327.1	6	12.8	36.0
OY292328.1	7	12.6	36.0
OY292329.1	8	12.45	36.0
OY292330.1	9	12.33	36.5
OY292331.1	10	12.2	36.0
OY292332.1	11	12.2	36.0
OY292333.1	12	11.81	36.0
OY292334.1	13	11.77	36.5
OY292335.1	14	11.6	36.5
OY292336.1	15	11.49	36.0
OY292337.1	16	11.42	36.5
OY292338.1	17	11.09	36.5
OY292339.1	18	10.54	37.0
OY292340.1	19	10.49	37.0
OY292341.1	20	10.26	36.5
OY292342.1	21	9.96	37.0
OY292343.1	22	9.22	36.5
OY292344.1	23	8.94	37.5
OY292345.1	24	8.69	36.5
OY292346.1	25	8.36	37.0
OY292347.1	26	7.95	37.0
OY292348.1	27	6.3	38.0
OY292349.1	28	6.01	39.0
OY292350.1	29	5.77	40.0
OY292351.1	30	5.16	40.0
OY292321.1	Z	15.08	36.5
OY292352.1	MT	0.02	19.0

The estimated Quality Value (QV) of the final assembly is 68.1 with *k*-mer completeness of 100.0%, and the assembly has a BUSCO v5.3.2 completeness of 99.1% (single = 98.9%, duplicated = 0.2%), using the lepidoptera_odb10 reference set ( *n* = 5,286).

Metadata for specimens, barcode results, spectra estimates, sequencing runs, contaminants and pre-curation assembly statistics are given at https://links.tol.sanger.ac.uk/species/542982.

## Genome annotation report

The *Heliothis peltigera* genome assembly (GCA_958496145.1) was annotated at the European Bioinformatics Institute (EBI) on Ensembl Rapid Release. The resulting annotation includes 17,323 transcribed mRNAs from 17,114 protein-coding genes ( Table 1; https://rapid.ensembl.org/Heliothis_peltigera_GCA_958496145.1/Info/Index).

## Methods

### Sample acquisition and nucleic acid extraction

A male *Heliothis peltigera* (specimen ID Ox002232, ToLID ilHelPelt1) was collected from Bratton, Somerset, UK (latitude 51.20, longitude –3.51) on 2022-06-20, using a light trap. The specimen was collected by Denise Wawman (University of Oxford) and identified by Liam Crowley (University of Oxford), and then snap-frozen on dry ice.

The workflow for high molecular weight (HMW) DNA extraction at the Wellcome Sanger Institute (WSI) includes a sequence of core procedures: sample preparation; sample homogenisation, DNA extraction, fragmentation, and clean-up. In sample preparation, the ilHelPelt1 sample was weighed and dissected on dry ice ( Jay *et al.*, 2023). Tissue from the thorax was homogenised using a PowerMasher II tissue disruptor ( Denton *et al.*, 2023a). HMW DNA was extracted using the Automated MagAttract v2 protocol ( Oatley *et al.*, 2023a). DNA was sheared into an average fragment size of 12–20 kb in a Megaruptor 3 system with speed setting 31 ( Bates *et al.*, 2023). Sheared DNA was purified by solid-phase reversible immobilisation ( Oatley *et al.*, 2023b): in brief, the method employs a 1.8X ratio of AMPure PB beads to sample to eliminate shorter fragments and concentrate the DNA. The concentration of the sheared and purified DNA was assessed using a Nanodrop spectrophotometer and Qubit Fluorometer and Qubit dsDNA High Sensitivity Assay kit. Fragment size distribution was evaluated by running the sample on the FemtoPulse system.

Protocols developed by the WSI Tree of Life laboratory are publicly available on protocols.io ( Denton *et al.*, 2023b).

### Sequencing

Pacific Biosciences HiFi circular consensus DNA sequencing libraries were constructed according to the manufacturers’ instructions. DNA sequencing was performed by the Scientific Operations core at the WSI on a Pacific Biosciences SEQUEL II instrument. Hi-C data were also generated from head tissue of ilHelPelt1 using the Arima2 kit and sequenced on the Illumina NovaSeq 6000 instrument.

### Genome assembly, curation and evaluation

Assembly was carried out with Hifiasm ( Cheng *et al.*, 2021) and haplotypic duplication was identified and removed with purge_dups ( Guan *et al.*, 2020). The assembly was then scaffolded with Hi-C data ( Rao *et al.*, 2014) using YaHS ( Zhou *et al.*, 2023). The assembly was checked for contamination and corrected as described previously ( Howe *et al.*, 2021). Manual curation was performed using gHiGlass ( Kerpedjiev *et al.*, 2018) and PretextView ( Harry, 2022). The mitochondrial genome was assembled using MitoHiFi ( Uliano-Silva *et al.*, 2023), which runs MitoFinder ( Allio *et al.*, 2020) or MITOS ( Bernt *et al.*, 2013) and uses these annotations to select the final mitochondrial contig and to ensure the general quality of the sequence.

A Hi-C map for the final assembly was produced using bwa-mem2 ( Vasimuddin *et al.*, 2019) in the Cooler file format ( Abdennur & Mirny, 2020). To assess the assembly metrics, the *k*-mer completeness and QV consensus quality values were calculated in Merqury ( Rhie *et al.*, 2020). This work was done using Nextflow ( Di Tommaso *et al.*, 2017) DSL2 pipelines “sanger-tol/readmapping” ( Surana *et al.*, 2023a) and “sanger-tol/genomenote” ( Surana *et al.*, 2023b). The genome was analysed within the BlobToolKit environment ( Challis *et al.*, 2020) and BUSCO scores ( Manni *et al.*, 2021; Simão *et al.*, 2015) were calculated.

Table 3 contains a list of relevant software tool versions and sources.

**Table 3.** Software tools: versions and sources.

Software tool	Version	Source
BlobToolKit	4.2.1	https://github.com/blobtoolkit/blobtoolkit
BUSCO	5.3.2	https://gitlab.com/ezlab/busco
Hifiasm	0.16.1-r375	https://github.com/chhylp123/hifiasm
HiGlass	1.11.6	https://github.com/higlass/higlass
Merqury	MerquryFK	https://github.com/thegenemyers/MERQURY.FK
MitoHiFi	3	https://github.com/marcelauliano/MitoHiFi
PretextView	0.2	https://github.com/wtsi-hpag/PretextView
purge_dups	1.2.5	https://github.com/dfguan/purge_dups
sanger-tol/genomenote	v1.0	https://github.com/sanger-tol/genomenote
sanger-tol/readmapping	1.1.0	https://github.com/sanger-tol/readmapping/tree/1.1.0
YaHS	1.2a.2	https://github.com/c-zhou/yahs

### Genome annotation

The BRAKER2 pipeline ( Brůna *et al.*, 2021) was used in the default protein mode to generate annotation for the *Heliothis peltigera* assembly (GCA_958496145.1) in Ensembl Rapid Release at the EBI.

### Wellcome Sanger Institute – Legal and Governance

The materials that have contributed to this genome note have been supplied by a Darwin Tree of Life Partner. The submission of materials by a Darwin Tree of Life Partner is subject to the **‘Darwin Tree of Life Project Sampling Code of Practice’**, which can be found in full on the Darwin Tree of Life website here. By agreeing with and signing up to the Sampling Code of Practice, the Darwin Tree of Life Partner agrees they will meet the legal and ethical requirements and standards set out within this document in respect of all samples acquired for, and supplied to, the Darwin Tree of Life Project. 

Further, the Wellcome Sanger Institute employs a process whereby due diligence is carried out proportionate to the nature of the materials themselves, and the circumstances under which they have been/are to be collected and provided for use. The purpose of this is to address and mitigate any potential legal and/or ethical implications of receipt and use of the materials as part of the research project, and to ensure that in doing so we align with best practice wherever possible. The overarching areas of consideration are:

• Ethical review of provenance and sourcing of the material

• Legality of collection, transfer and use (national and international) 

Each transfer of samples is further undertaken according to a Research Collaboration Agreement or Material Transfer Agreement entered into by the Darwin Tree of Life Partner, Genome Research Limited (operating as the Wellcome Sanger Institute), and in some circumstances other Darwin Tree of Life collaborators.

## Data Availability

European Nucleotide Archive: *Heliothis peltigera* (bordered straw). Accession number PRJEB62179; https://identifiers.org/ena.embl/PRJEB62179 ( Wellcome Sanger Institute, 2023). The genome sequence is released openly for reuse. The *Heliothis peltigera* genome sequencing initiative is part of the Darwin Tree of Life (DToL) project. All raw sequence data and the assembly have been deposited in INSDC databases. Raw data and assembly accession identifiers are reported in Table 1.

## Author information

Members of the University of Oxford and Wytham Woods Genome Acquisition Lab are listed here: https://doi.org/10.5281/zenodo.7125292.

Members of the Darwin Tree of Life Barcoding collective are listed here: https://doi.org/10.5281/zenodo.4893703.

Members of the Wellcome Sanger Institute Tree of Life Management, Samples and Laboratory team are listed here: https://doi.org/10.5281/zenodo.10066175.

Members of Wellcome Sanger Institute Scientific Operations: Sequencing Operations are listed here: https://doi.org/10.5281/zenodo.10043364.

Members of the Wellcome Sanger Institute Tree of Life Core Informatics team are listed here: https://doi.org/10.5281/zenodo.10066637.

Members of the Tree of Life Core Informatics collective are listed here: https://doi.org/10.5281/zenodo.5013541.

Members of the Darwin Tree of Life Consortium are listed here: https://doi.org/10.5281/zenodo.4783558.
