# Supplementary material for: The genome sequence of the Bordered Straw, Heliothis peltigera (Denis & Schiffermüller) 1775
Source: Wellcome Open Res. 2024 Apr 15;9:195. [Version 1] doi: 10.12688/wellcomeopenres.21221.1 (PMC11318084; doi:10.12688/wellcomeopenres.21221.1)
Supplement: Peer review history

# Reviewer report: Reviewer response for version 1

**DOI:** 10.21956/wellcomeopenres.23468.r90165

**Published:** 2024-08-09

**Authors:** Qiye Li

**Affiliations:** 1 BGI Research, Beijing, China; 2 College of Life Sciences, University of Chinese Academy of Sciences, Beijing, China

In this study, Wawman *et al.* present the first chromosome-scale genome assembly of the Bordered Straw ( *Heliothis peltigera* ) based on PacBio HiFi long-read and Hi-C long-range sequencing data. The contiguity, completeness and accuracy of the genome assembly are excellent. Together with the protein-coding genes annotated for the genome, this study provides a valuable dataset for the community. However, I do have some suggestions that may help improve the manuscript.

 1. In the last paragraph of Background, the authors claim that they present a “chromosomally complete genome sequence” for *Heliothis peltigera* . Given that there are still some gaps inside the chromosomes, and that the presence of telomeres on chromosomal ends has not been ascertained, I would suggest avoiding emphasizing the genome sequence is complete. Instead, “near-complete” is a more cautious statement.

 2. The authors state that “The Z chromosome was identified based on synteny with *Craniophora ligustri* (GCA_905163465.1) (Boyes et al., 2023).” Given that sex chromosome transitions are quite common in some animal lineages, could the authors explain a little further in the manuscript why this is workable? For example, is that because the sex chromosomes are highly conserved across Noctuidae species? This information is not clear to the readers.

 3. All the software tools used for building and assessing the genome assembly have been provided in methods and table 3. But to allow replication by others, the parameters of each tool are required as well. Even if default parameters are used, it is worthy to mention somewhere in the manuscript. I suggest that the authors may provide the comments of parameters used for each tool in table 3, especially when some tools are run with default settings, but some are not.

 4. The protein-coding genes are annotated for the genome assembly, which are valuable for future studies. However, the completeness of the gene set is not assessed. At least, presenting the assessment for the gene set using BUSCO will be informative.

**Some minor comments:**

 1. The first sentence of Background, “The Bordered Straw *Heliothis peltigera* is a Noctuid moth, but although…… to become darker (Waring et al., 2017)”, is quit long and a bit hard to follow. Perhaps, the authors can break it into two or more sentences?

 2. The Latin name of *Heliothis peltigera* has been spelled out for multiple times in the main text. Please consider using “ *H. peltigera* ” after the first time of spelling out.

Are sufficient details of methods and materials provided to allow replication by others?

Partly

Is the rationale for creating the dataset(s) clearly described?

Yes

Are the datasets clearly presented in a useable and accessible format?

Yes

Are the protocols appropriate and is the work technically sound?

Yes

Reviewer Expertise:

Biodiversity genomics

I confirm that I have read this submission and believe that I have an appropriate level of expertise to confirm that it is of an acceptable scientific standard.

# Reviewer report: Reviewer response for version 1

**DOI:** 10.21956/wellcomeopenres.23468.r90157

**Published:** 2024-08-07

**Authors:** Savarimuthu Ignacimuthu, Sivasankaran Kuppusamy

**Affiliations:** 1 St. Xavier's College, Interdisciplinary Research Centre in Biology, Xavier Research Foundation, Manonmaniam Sundaranar University, Thirunelveli, TN, India; 2 Entomology Research Institute, Loyola College, Chennai, Tamil Nadu, India

**Review Report**

 The authors have sequenced the genome of *Heliothis peltigera* (Denis & Schiffermüller) 1775 with Next Generation Sequence techniques. A total of 332.8 megabases in span were assembled through the genome assemble. Totally 17,114 protein-coding genes and 17,323 number of gene transcripts were observed through the annotation. Authors have utilized appropriate techniques for DNA isolation, library construction for sequencing and used proper software for assembly and annotation.  

**Comment 1:**

 The author’s name of the species with year can be included in the first sentence of the background. “The Bordered Straw *Heliothis peltigera* (Denis & Schiffermüller) 1775 is a Noctuid moth…”

 There is confusion in this sentence “but although it has the characteristic thick body and wing-shapeseen in this family”. Please delete the sentence or can be modified.

**Comments 2:**

 Authors have used the word kidney instead of reniform in the background of the species. Actually, it’s a kidney shaped or renal shaped (reniform) marking not kidney. So, the second sentence of the background can be changed as

 “It is usually more distinctly marked than similar species with a bold grey reniform mark and diagnostic dark diffuse blotches on the leading edge of the forewing near the reniform and adjacent to the darker band on the outer edge: the similar Scarce Bordered Straw or Old World Bollworm *Helicoverpa armigera* lacks both of these diagnostic markings, and Eastern Bordered Straw *Heliothis nubigera* has only a smaller more diffuse marking near the reniform, with a paler ground colour and three or more distinct small dots near the outer edge (Skinner & Wilson, 2009; Waringet al., 2017)”.

**Comment 3:**

 Authors have given the genus name *Heliothis peltigera* full form throughout the article. For the first time the genus name can be given in full form then subsequently can be given in short form like *H. peltigera*

**Comment 4:**

 The second paragraph first sentence fourth line “remove the comma (,) after and…..

**Comment 5:**

 Total length of the mitochondrial genome sequence must be provided in the text.

 Above all, I confirm that the manuscript meets the necessary scientific standard and is suitable for indexing"

Are sufficient details of methods and materials provided to allow replication by others?

Yes

Is the rationale for creating the dataset(s) clearly described?

Yes

Are the datasets clearly presented in a useable and accessible format?

Yes

Are the protocols appropriate and is the work technically sound?

Yes

Reviewer Expertise:

Molecular biology

We confirm that we have read this submission and believe that we have an appropriate level of expertise to confirm that it is of an acceptable scientific standard.
